# P-679. Invasive pneumococcal pneumonia: is bacteremia the appropriate indication of severity?

**DOI:** 10.1093/ofid/ofaf695.892

**Published:** 2026-01-11

**Authors:** Paula Peyrani, Lindsay Grant, Mohammad Ali, Paul Palmer, Alejandro D Cane, Michael Bois, Jelena Vojicic

**Affiliations:** Pfizer, Inc, Collegeville, PA; Pfizer Inc., Collegeville, PA; Pfizer Inc., Collegeville, PA; Pfizer Vaccine Medical Development, Scientific & Clinical Affairs , Collegeville PA, Collegeville, PA; Pfizer, Collegeville, Pennsylvania; Pfizer, Collegeville, Pennsylvania; Pfizer Canada, Kirkland, QC, Canada

## Abstract

**Background:**

The most common presentation of pneumococcal disease in adults is pneumonia, which in approximately 10% presents as bacteremic pneumonia and is classified as invasive pneumococcal disease (IPD). There is conflicting data regarding the role of bacteremia in clinical outcomes of patients hospitalized with pneumococcal community-acquired pneumonia (CAP). The primary objective of this study was to compare in-hospital mortality rates in patients hospitalized with bacteremic vs non-bacteremic pneumococcal CAP.

**Figure:**
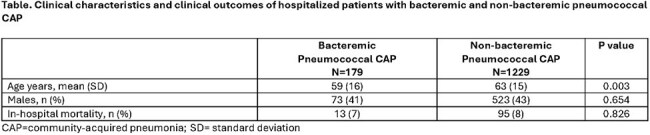

**Methods:**

This is a post-hoc analysis of 2 multicenter studies of adults ≥18 years of age hospitalized with CAP during 2014-2016 and 2019-2020 from multiple sites in the US. Urine was collected and tested by Pfizer’s serotype-specific urinary antigen detection assay, which detects 24 serotypes. Results from additional microbiological tests collected for clinical reasons were recorded. Patients were assigned to the bacteremic CAP group only if the blood culture grew *S. pneumoniae*. The non-bacteremic CAP group included *S. pneumoniae* positive patients by any other test. Clinical history was obtained from the medical chart. In-hospital mortality was defined as death of any cause within 30 days after admission.

**Results:**

Among 14,160 adults with radiologically confirmed CAP, 1408 (10.0%) had *S. pneumoniae* identified in at least 1 test. Bacteremia was identified in 13% (n=179) of the patients with pneumococcal pneumonia and a serotype was identified in 84% (n=1,381) of these patients. Basic demographics and in-hospital mortality rates are described in Table 1.

**Conclusion:**

In-hospital mortality did not differ between bacteremic and non-bacteremic CAP in this study. These data suggest that severe pneumococcal CAP may not be defined by the presence of bacteremia. Further research evaluating the pathophysiology and consequences of bacteremia in pneumococcal CAP is merited.

**Disclosures:**

Paula Peyrani, MD, Pfizer, Inc: Employee|Pfizer, Inc: Stocks/Bonds (Public Company) Lindsay Grant, PhD, MPH, Pfizer: Employee|Pfizer: Stocks/Bonds (Private Company) Mohammad Ali, PhD, Pfizer Vaccines: Employee|Pfizer Vaccines: Stocks/Bonds (Public Company) Paul Palmer, PhD, Pfizer Inc: Employee|Pfizer Inc: Stocks/Bonds (Public Company) Alejandro D. Cane, MD, PhD, Pfizer Inc.: All authors are employees of Pfizer Inc. and may hold stock and/or stock options of Pfizer Inc. Michael Bois, PhD, M(ASCP), PFIZER: Stocks/Bonds (Public Company) Jelena Vojicic, MD, Pfizer Inc.: Employee|Pfizer Inc.: Stocks/Bonds (Public Company)|Pfizer Inc.: Stocks/Bonds (Public Company)

